# Auxeticity as a Mechanobiological Tool to Create Meta-Biomaterials

**DOI:** 10.1021/acsabm.3c00145

**Published:** 2023-06-15

**Authors:** Ebrahim Yarali, Amir A. Zadpoor, Urs Staufer, Angelo Accardo, Mohammad J. Mirzaali

**Affiliations:** † Department of Biomechanical Engineering, Faculty of Mechanical Maritime and Materials Engineering, Delft University of Technology (TU Delft), Mekelweg 2, 2628 CD Delft, The Netherlands; ‡ Department of Precision and Microsystems Engineering, Faculty of Mechanical Maritime and Materials Engineering, Delft University of Technology (TU Delft), Mekelweg 2, 2628 CD Delft, The Netherlands

**Keywords:** Meta-biomaterials, Poisson’s ratio, auxeticity, bone tissue engineering, cell response, mechanobiology, additive manufacturing, 4D printing

## Abstract

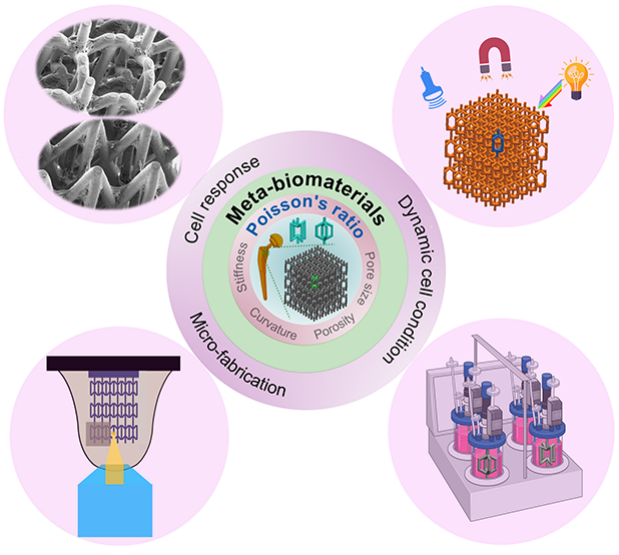

Mechanical and morphological design parameters, such as stiffness or porosity, play important roles in creating orthopedic implants and bone substitutes. However, we have only a limited understanding of how the microarchitecture of porous scaffolds contributes to bone regeneration. Meta-biomaterials are increasingly used to precisely engineer the internal geometry of porous scaffolds and independently tailor their mechanical properties (e.g., stiffness and Poisson’s ratio). This is motivated by the rare or unprecedented properties of meta-biomaterials, such as negative Poisson’s ratios (i.e., auxeticity). It is, however, not clear how these unusual properties can modulate the interactions of meta-biomaterials with living cells and whether they can facilitate bone tissue engineering under static and dynamic cell culture and mechanical loading conditions. Here, we review the recent studies investigating the effects of the Poisson’s ratio on the performance of meta-biomaterials with an emphasis on the relevant mechanobiological aspects. We also highlight the state-of-the-art additive manufacturing techniques employed to create meta-biomaterials, particularly at the micrometer scale. Finally, we provide future perspectives, particularly for the design of the next generation of meta-biomaterials featuring dynamic properties (e.g., those made through 4D printing).

## Introduction

Three-dimensional (3D) lattice structures have been widely studied for the design of orthopedic implants that are used for complex bone reconstructions. The success of designing these porous implants relies on several factors, including the mechanical properties of the constituting materials (e.g., stiffness), their geometrical features at the microscale (e.g., pore geometry), and their local surface characteristics.^1^ All these design factors must be considered simultaneously to adequately mimic the (micro)environment of the (bony) tissue and facilitate the tissue integration process (i.e., osseointegration).

“Stress-shielding” may occur at the bone–implant interfaces if the mechanical properties of the implanted biomaterial (e.g., metal- or ceramic-based materials characterized by high stiffness) do not match those of the host tissue particularly when the implant is stiffer than the surrounding tissue, causing its local deformations to be smaller than they would naturally be. According to Wolff’s law, this phenomenon can result in bone resorption and, eventually, aseptic loosening.^2^

From a microarchitectural viewpoint, implants and scaffolds need to be porous for several reasons: (*i*) to effectively mimic the morphology of the bone;^3^ (*ii*) to facilitate mass transport within the scaffolds/implants, enabling the delivery of nutrients and oxygen to the cells residing in the scaffolds;^4^ and (*iii*) to replicate the stiffness of the native bone which ranges between 0.2 and 20 GPa.^5^

The response of bone cells to biomaterials (e.g., cell adhesion, proliferation, and differentiation) is influenced by the geometry of such porous structures, including pore shape, pore size, porosity, (local) surface curvatures, and surface nanopatterning. The effects of some of the geometrical parameters, such as porosity,^6^ surface curvature,^7^ and surface nanopatterning,^8^ on one hand and those of the elastic modulus^9^ on the other hand have been extensively studied. However, the effects of some other design parameters, including the Poisson’s ratio, on the bone regeneration process in general and bone cell response in particular remain elusive. Any such effects can either result from the mechanical behavior associated with auxeticity or be a direct consequence of the specific shape of the unit-cells used for creating auxetic behavior in such architected biomaterials (e.g., the re-entrant unit-cell). In both cases, mechanobiological pathways are expected to be responsible for regulating the effects of auxeticity on the bone regeneration process.

To gain a better understanding of how various geometrical and mechanical factors influence the cell response and bone tissue regeneration process, it is important to separate the various effects from each other as much as possible and study them in isolation. One effective approach for tuning, controlling, and decoupling mechanical and morphological properties is the use of a class of engineered architected materials known as mechanical metamaterials. The distinct, unusual properties of these materials at the mesoscale originate from their (geometrical) design at the microscale.^2,10−11^ Among the unusual properties of mechanical metamaterials is auxeticity or a negative value of the Poisson’s ratio (NPR). Due to their microarchitectural designs, such as the geometry of their unit-cells, mechanical metamaterials with NPR expand transversely when stretched longitudinally. In biomedical applications, biocompatible materials can be employed to create multiphysics metamaterials, which are defined as meta-biomaterials.^2,12,300^ To rationally design meta-biomaterials with controlled mechanical and morphological properties, as well as adequate mechanical strength, various methods, such as (topology) optimization,^11^ artificial intelligence (e.g., machine learning),^13,400^ analytical models,^14^ and finite element analyses^15^ have been employed, depending on the specific requirements of the application at hand. These techniques utilize mathematical algorithms to optimize the material’s configuration, predict its mechanical properties, and stimulate its behavior under different loading conditions. Combining these methods provides a robust approach to design advanced meta-biomaterials tailored to meet diverse biomedical needs.^6000^

Interestingly, auxetic behavior has been frequently observed in biological materials, including hard tissues,^16^ soft tissues,^17^ and cells,^18^ highlighting its importance as a mechanobiological design tool for creating biomimetic meta-biomaterials. Recent studies have also shown that auxeticity in meta-biomaterials can modulate cell differentiation and proliferation^19−22^ and may guide the alignment, orientation, and migration of cells (e.g., fibroblast).^20^ Moreover, the rational design of the microarchitecture of meta-biomaterials can enhance the mechanical fixation and longevity of meta-biomaterial-based implants as compared to their conventional counterparts.^23^ Finding an optimal value for the Poisson’s ratio while decoupling it from other mechanical (e.g., stiffness) and geometrical parameters (e.g., porosity) presents a significant challenge. The entanglement of these parameters makes it challenging to study the individual effects of a specific parameter on the biological response of 3D models. This gap in the literature underscores the need for further research to assess the true effects of auxeticity on cell response at different length scales.

Furthermore, since the geometry of the unit-cell in meta-biomaterials changes under (mechanical) loading conditions,^7000,8000^ a better understanding of the role of auxeticity in interactions with living cells is required. This understanding will significantly contribute to the design of meta-biomaterials and their ability to facilitate tissue regeneration. We have, therefore, dedicated a section of this review to the mechanobiological studies of meta-biomaterials in dynamic cell microenvironments.

This article reviews the currently existing evidence regarding the ways in which auxetic behavior influences the performance of biomaterials. We critically discuss the potential of auxeticity as a design tool for the development of the next generation of meta-biomaterials and summarize the recent literature on the consequences of (non)auxetic behavior on the responses of living cells. To this end, we propose novel design approaches and testing methods to incorporate the effects of the Poisson’s ratio into the design of meta-biomaterials for future research. Furthermore, we review advanced additive manufacturing and 4D printing techniques that can be used for creating meta-biomaterials with time-dependent properties.

## Auxeticity in Biological Materials

Auxeticity is found in soft tissues, hard tissues, organs, and cells. Examples of hard tissues exhibiting NPR include trabecular bone^16^ and the annulus fibrosus of the intervertebral disc.^21,24,500^ Soft tissues showing auxetic behavior include cat skin,^25^ salamander skin,^26^ arterial endothelium^27,28^ (under both wall shear stresses and cyclic circumferential strain induced by blood flow), cow teat skin,^29^ arteries,^28,30^ and tendons.^17^ In addition, some evidence of auxetic behavior has been found in living cells, such as embryonic epithelia^31,600^ and the nuclei of embryonic stem cells.^18^

**Figure 1:**
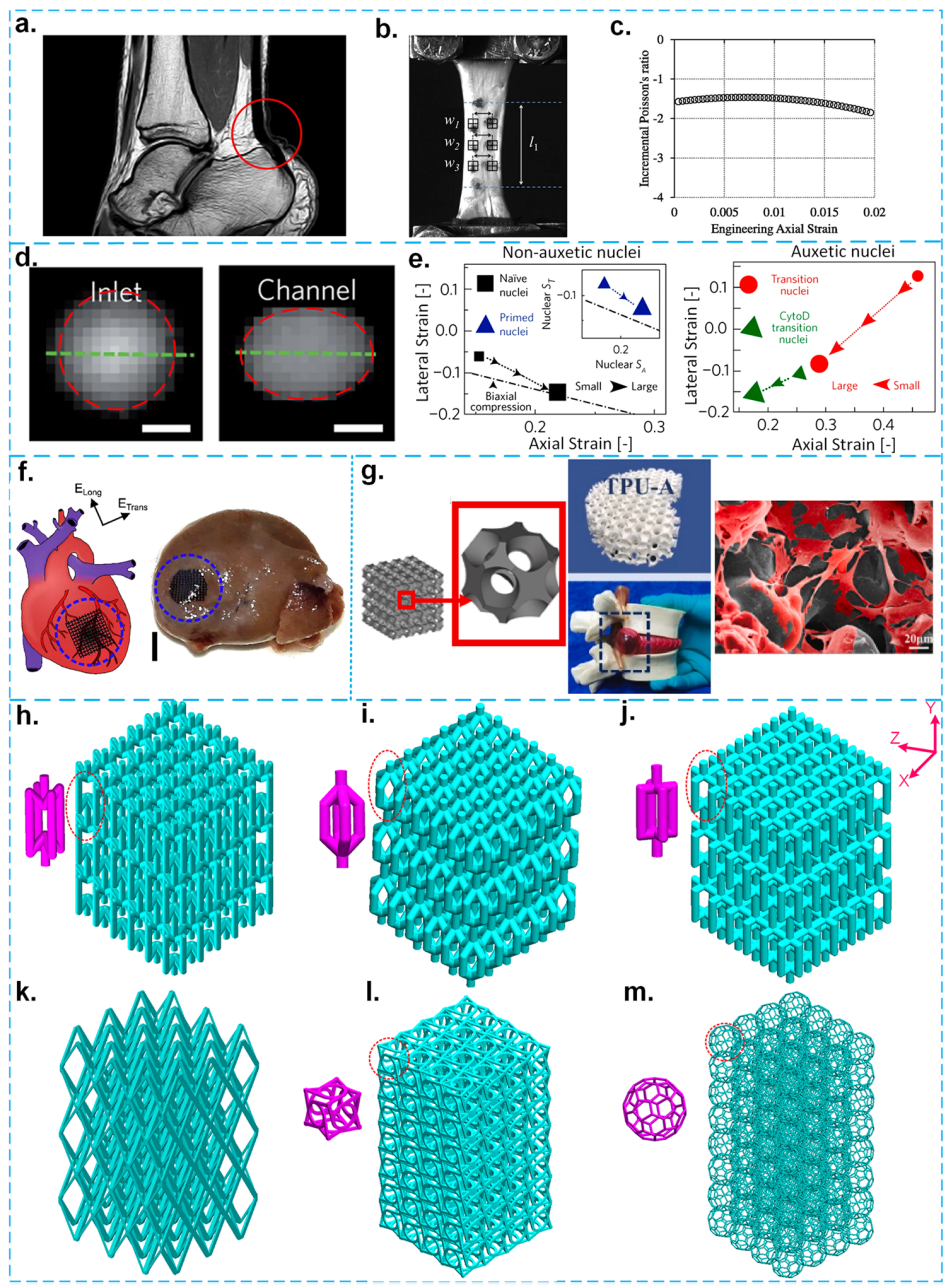
a–c. Tendon is an example of a soft tissue showing auxetic behavior. Adapted with permission from ref (17). Copyright 2015 Elsevier. a. Some MRI images of the human tendon. b. This has been observed in an MRI image of the human tendon that expands under stretching in vivo. c. Some ex vivo results of the uniaxial testing of the human Achilles tendon showing the dependency of the Poisson’s ratio on the applied axial strain. d,e. Auxeticity in the nuclei of embryonic stem cells. Adapted with permission from ref (18). Copyright 2014 Springer Nature. d. Epifluorescence images of a cross-section of the nuclei of a cell before and after entering a microfluidic channel. e. The variation of the lateral strain (i.e., *S*_T_) with the axial strain (i.e., *S*_A_) for both non-auxetic and auxetic nuclei. f. A schematic drawing of an auxetic patch and a representative image of the auxetic patch implemented in a rat after 2 weeks;^9^ the scale bar shows 2 mm. g. The in vivo implementation of an auxetic surface-based meta-scaffold into the LDH of a rabbit.^32^ (left) A schematic drawing of the NPR meta-biomaterial and its constituting unit-cell; (middle) testing the mechanical performance of the NPR meta-biomaterial using a commercially available LDH model; (right) the SEM image of nucleus pulposus cells when adhered to an NPR meta-biomaterial. h–m. Some examples of strut-based meta-biomaterials with h., NPR; i., PPR; j., nearly ZPR; and k., transversely isotropic properties. Reproduced with permission from ref (55). Copyright 2020 Elsevier. l. Chiral metamaterials (bending-dominated). m. Isotropic buckyball meta-biomaterial.

To measure the auxetic behavior of these biological materials, different techniques, such as imaging, computational modeling, and in vitro mechanical testing, have been employed. For example, the auxetic behavior of trabecular bone has been studied by performing in vitro experiments on the human tibia under triaxial compressive loading. The auxeticity in the spongy parts of such bones has been demonstrated by calculating the material constants of a transversely isotropic model via computational models.^16^ It should, however, be noted that the values of the Poisson’s ratio in biological materials may depend on the level of the applied strains or the aspect ratios of the tested tissue specimens. For example, in cow teat skin, NPR is only reported for specimens with a specific range of length to width ratios (i.e., 1.4–2.45) and under certain levels (35%) of applied strains.^29^ in vivo and ex vivo experiments have been performed on tendon specimens taken from several species, such as human peroneus brevis, human Achilles tendons, and deep flexor tendons (pig and sheep) to study their auxetic behavior (Figure 1a–c).^17^ The Poisson’s ratio has been measured using nondestructive medical imaging techniques (e.g., magnetic resonance imaging (MRI)) for in vivo conditions and mechanical testing for ex vivo^17^ conditions (Figure 1c). Regarding the auxeticity in the nuclei of embryonic stem cells during the differentiation process,^18^ it has been found that the cross-section of the nuclei contracts under compressive loading.^18^ Moreover, the stiffness of the nuclei has been found to increase under compressive loading^18^ (Figure 1d and e). These observations have been verified using fluorescent optical microscopy and scanning electron microscopy (SEM), as well as by measuring local forces using atomic force microscopy.^18^

There are also studies employing auxeticity in the design and implementation of medical devices. For example, in ex vivo studies, auxetic cardiac patches have been used to mimic the native heart movements against myocardial infraction (Figure 1f).^9^ Furthermore, an auxetic meta-biomaterial has been successfully implemented for the treatment of lumbar disc herniation (LDH) in an in vivo rabbit model (Figure 1g).^32^ Further review of such studies is, however, beyond the scope of this review article, as we will be only focusing on the in vitro mechanobiological behavior of meta-biomaterials.

There are several biological substances whose Poisson’s ratio is near zero (ZPR). These materials, therefore, exhibit no to little contraction or expansion when subjected to compression or tension. Examples of these biological materials are cartilage, cornea, and brain.^33,700^ Poisson’s ratio-driven meta-biomaterial designs (i.e., auxetic, zero, or non-auxetic) may, therefore, help in mimicking the properties of native tissues and facilitate the regeneration of tissues in vitro,^19−21,34^ ex vivo,^9^ and in vivo.^32^

## Auxeticity in Bone Tissue Engineering

### Poisson’s Ratio-Driven Mechanotransduction

The microenvironment sensed by cells is an important factor in bone tissue engineering. Geometry (e.g., surface curvature, pore shape), surface characteristics (roughness, cell-friendly coating), and the cell culture conditions (i.e., static or dynamic) are among the factors determining the microenvironment of cells. They affect cell–cell and cell–extracellular matrix (ECM) interactions, the plasma membrane, the cytoskeleton, and nuclear components through integrin-mediated force-feedback at adhesion sites.^7,35−37^ Cells are constantly exposed to various mechanical stimulations, both extracellular and intracellular, and can respond to changes in these forces through mechanotransductory pathways. These pathways involve the conversion of mechanical signals into biochemical signals that regulate cell behavior.^7,37,38^ This conversion is mediated by a range of specialized proteins and molecules, including integrins, focal adhesions (vinculin, paxillin), cytoskeletal elements, and signaling molecules. These components work together to orchestrate the formation of complex networks that can activate or inhibit various cellular pathways.^7,39^

Mechano-receptors, such as integrins, initiate mechano-sensation through physical bonding between the bone cells and loading via the ECM. The connection between mechanotransduction and cellular responses can be studied via both biological assays and computational tools.^40^ While there are numerous studies examining these processes in 2D environments, the mechanotransductory mechanisms associated with 3D meta-biomaterials remain largely elusive.^41^

Mechanical cues modulate the remodeling rate of the bony tissue and influence its regeneration.^35^ Cellular processes, such as adhesion, proliferation, differentiation, and gene expression are, therefore, affected by mechanical forces, in addition to biophysical cues, such as geometry and substrate stiffness. The above-mentioned microenvironmental factors can change the mechanical forces (e.g., stretching, compressive, and shear flow) that can alter the mechanobiology of cells^35^ (e.g., bone cells,^42^ epithelial cells^43^) through changes in the magnitude or rate of the loads experienced by the cells. For example, bone cells respond to compressive forces and produce biochemical cues, such as prostaglandin, that lead to the formation of new tissue through interactions between biomechanical and biochemical cues.^35,42^ Moreover, it has been shown that auxeticity plays an important role in how mechanotransductory events affect stem cells.^18,22,44,45^ Although there has been limited research exploring the role of the Poisson’s ratio in mechanotransduction, a recent study has examined its impact on the focal adhesion of embryonic fibroblasts using immunological staining of vinculin.^45^ The study compares two different 2D meta-biomaterials with positive and negative values of the Poisson’s ratios, and finds that both structures exhibit similar patterns of integrin marker expression, indicating that the Poisson’s ratio may not significantly impact integrin-mediated adhesions in 2D environments.^45^ However, further studies are needed to fully understand how the Poisson’s ratio and other mechanical properties of 3D meta-biomaterials affect mechanotransduction and cell behavior.

Understanding the interplay between mechanical properties and cellular behavior is crucial for the development of advanced meta-biomaterials with tailored properties for use in various biomedical applications. Further research in this area could inform the design of these materials and improve their performance in tissue engineering and regenerative medicine.

The rational design of microarchitectures of meta-biomaterials will, thus, allow for tuning the local deformations developed in meta-biomaterials in response to globally applied deformations and enable the on-demand generation of mechanotransductory cues for controlling bone modeling or remodeling processes. The links between physical cues (e.g., materials properties, stiffness),^46^ surface (bio)functionalization,^47^ and geometry (e.g., curvature^7^) on one hand and biochemical signaling of cells on the other have been extensively studied. Hence, we only focus on the role of auxeticity in the mechanobiological response of meta-biomaterials, particularly for bone tissue engineering purposes.

### Meta-Biomaterials and Their Interactions with Living Cells

The emergence of meta-biomaterials has provided unparalleled opportunities in expanding the design space of biomedical devices. Meta-biomaterials pave the way for establishing optimal architecture-property-functionality relationships so that multifunctional biomedical devices (e.g., orthopedics implants) can be developed.

Mechanical metamaterials are composed of several building blocks or unit-cells that can be arranged in an ordered or disordered manner. This makes their effective properties different from those of the base materials from which they are made and directly links them to the design of their microarchitecture. Examples of these unusual properties are ultrastiffness, ultralight weight (i.e., the ratio of the elastic modulus to density),^48^ sequential shape change,^49^ negative compressibility,^50^ and NPR (or auxeticity)^51^ in which the effective shear modulus is larger than the bulk modulus.^52^ Here, we only focus on the auxetic behavior of meta-biomaterials and discuss the methodologies proposed in the past for tuning this specific property.

The rational design of the microarchitectures of meta-biomaterials is the first step in adjusting their auxetic behaviors. In this regard, meta-biomaterials can be divided into two main categories, namely, strut-based and sheet-based meta-biomaterials. Although the Poisson’s ratio can be tuned from negative to positive in sheet-based meta-biomaterials too,^53^ there is currently limited information available regarding the interaction of sheet-based auxetic meta-biomaterials with living cells.^32^ We will, therefore, focus on strut-based meta-biomaterials and their mechanobiological responses. Moreover, cell culture conditions may play an important role in determining the response of cells to auxetic meta-biomaterials. Therefore, in the following sections, we provide an overview of the response of cells to strut-based meta-biomaterials under both static and dynamic cell culture conditions.

#### Meta-Biomaterials under Static Conditions

From a mechanical properties viewpoint, strut-based meta-biomaterials can be divided into two main subcategories, namely, stretch-dominated and bending-dominated.^52^ The parameter that determines whether a lattice structure is bending-dominated or stretch-dominated is the Maxwell number which is related to the average number of struts connecting to a specific node.^54,800^ In general, the higher the degree of connectivity, the higher the mechanical properties of the structure. From a lateral deformation viewpoint, however, the range of the Poisson’s ratio is wider in bending-dominated structures than in stretch-dominated structures. As such, the effects of auxeticity are more central in bending-dominated lattice structures. Figure 1h–m shows several examples of strut-based meta-biomaterials with different properties (e.g., a wide spread of Poisson’s ratios from negative to positive values). In such meta-biomaterials, the Poisson’s ratio depends on the angle between the struts, the width and height of the unit-cells, and the aspect ratio of the struts. The Poisson’s ratio of meta-biomaterials can, therefore, be adjusted within the thermodynamically admissible range of the Poisson’s ratio for isotropic materials (i.e., −1 to 0.5). Covering such a broad range of Poisson’s ratios is impossible within the realm of conventional materials. Moreover, the Poisson’s ratio of strut-based meta-biomaterials can be tuned to be either different or the same in various directions. For example, the Poisson’s ratio in two specific planes *zy* and *xy* (i.e., *v*_*xy*_ and *v*_*zy*_) can be designed to be equal (Figure 1h–j). In the meta-biomaterial depicted in Figure 1k, however, the Poisson’s ratios are different in different directions, as this transversely isotropic meta-biomaterial exhibits an NPR in one plane and a positive Poisson’s ratio (PPR) in another plane.^55^ Living cells may, therefore, experience different Poisson’s ratios or different (global or local) deformation regimes in different planes when interacting with such architected biomaterials.

There are several in vitro studies in the literature studying the mechanobiological properties of strut-based meta-biomaterials (either in 2D or 3D) with different Poisson’s ratios using different cell types (e.g., fibroblasts, osteoblasts, chondrocytes, and myoblasts).^19,22,30,45,56^ From the mechanical design viewpoint, however, the effects of the Poisson’s ratio are often coupled with those of microarchitectural design and mechanical properties (e.g., stiffness, pore size, porosity, and strut thickness). Given the fact that these properties are inter-related, changing the Poisson’s ratio without affecting the other parameters is extremely challenging.^22,56^ There is, therefore, not much evidence yet as to what the isolated effects of auxetic behavior are on cell response, making it difficult to determine whether auxetic materials are superior to non-auxetic biomaterials (i.e., structures with PPR or ZPR) in terms of cell differentiation and proliferation.

In addition to the shape of individual unit-cells, the dimensions of unit-cells play an important role in determining the biological response of meta-biomaterials. There is always a trade-off between the size of living biological cells and the feature size of the meta-biomaterial (e.g., pore size). If the pore size of the meta-biomaterial is significantly smaller than the size of a single cell, the cell growth inside such a microscale meta-biomaterial may be compromised.^21^ This is due to potential disturbance in mass transport. In mesoscale meta-biomaterials, however, the feature size of the lattice structure can be larger than the size of a single living cell (e.g., 1000 μm > pore size >100 μm), allowing cells to easily penetrate into the internal pores of the lattice structure. In such cases, the auxetic behavior can influence cell adhesion, cell proliferation, and cell differentiation.^20^ Moreover, in both micro- and mesoscale meta-biomaterials, cell alignment and migration can be influenced by auxeticity.^20^

**Figure 2:**
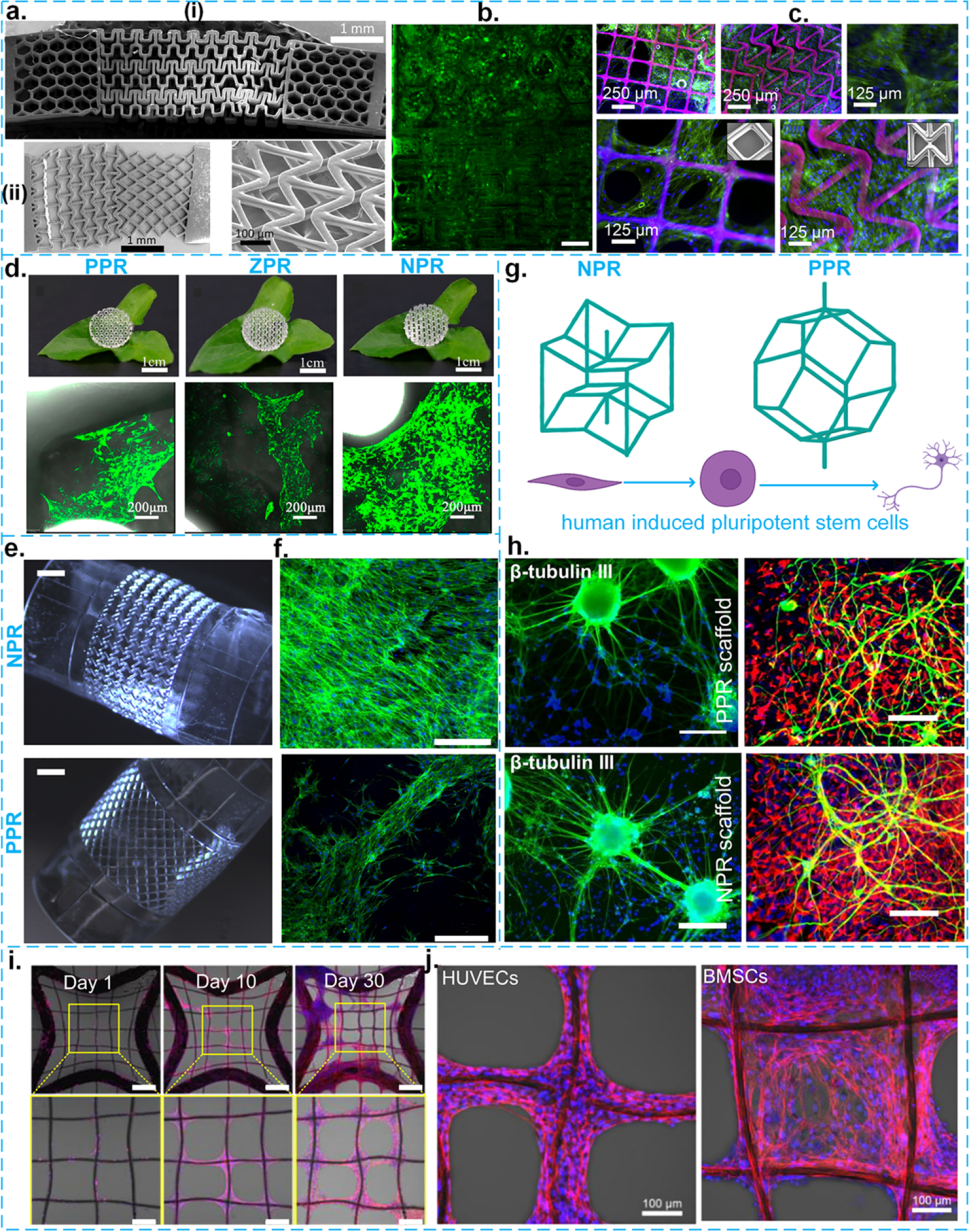
Some examples of cell responses to strut-based meta-biomaterials. a. Some examples of two different 2D hybrid meta-biomaterials and their SEM images: (a–i) Millimeter-scale 3D-printed meta-biomaterial made from polyaliphatic urethane acrylate with isobornyl acrylate (PAUA/IBOA) fabricated by using a stereolithography (SLA) technique (more specifically, dynamic optical projection stereolithography). Adapted with permission from ref (57). Copyright 2017 Elsevier. (a–ii) Millimeter-scale 3D-printed meta-biomaterials fabricated by a custom-made SLA technique from poly(ethylene glycol) diacrylate (PEGDA). Adapted with permission from ref (58). Copyright 2012 Elsevier. b. Fluorescent images of PPR and NPR regions of hybrid meta-biomaterials seeded with fibroblast cells after 3 weeks of cell culture. Adapted with permission from ref (57). Copyright 2017 Elsevier. The scale bar shows 250 μm. c. Fluorescent images showing F-actin and nuclei of the hybrid meta-biomaterial seeded with hMSCs. Adapted with permission from ref (58). Copyright 2012 Elsevier. d. The effects of three types of 2D meta-biomaterials with a positive, negative, and zero Poisson’s ratios (top side) on the proliferation of MSCs (bottom side).^19^ e and f. The interaction of hTMSCs with 2.5D cylindrical meta-biomaterials:^30^ e. Some optical microscopy images of NPR and PPR meta-biomaterials, which were 3D printed by SLA from PEGDA polymer. f. Confocal optical microscopy images of F-actin and nuclei of hTMSC; scale bar shows 300 μm. g and h. The response of mouse ESCs and hiPSCs to 3D strut-based meta-biomaterials made through a multistep thermomechanical fabrication technique from polyurethane foams. Adapted with permission from ref (56). Copyright 2018 John Wiley and Sons. Reproduced with permission from ref (22). Copyright 2017 Elsevier. g. The configurations of two 3D meta-biomaterials with PPR and NPR while being in contact with hiPSCs. Adapted with permission from ref (56). Copyright 2018 John Wiley and Sons. h. (left) Fluorescent images of the expression of the β-tubulin III marker of mouse ESCs within both PPR (upper row) and NPR scaffolds (lower row). Reproduced with permission from ref (22). Copyright 2017 Elsevier. The scale bar shows 200 μm. h. (right) Some fluorescent images of the expression of the neural markers (Hoechst, Nestin, and β-tubulin III) of the human iPSK3 cells within both PPR (upper row) and NPR scaffolds (lower row). Reproduced with permission from ref (22). Copyright 2017 Elsevier. The scale bar is 100 μm. Blue, red, and green show Hoechst, Nestin, and β-tubulin III, respectively. i and j. 2D multiscale NPR meta-biomaterials made through melt electrowriting (MEW). Adapted with permission from ref (60). Copyright 2021 Elsevier. i. Some fluorescent images of F-actin and nuclei of BMSCs on days 1, 10, and 30, with different magnifications. The scale bar is 200 μm. j. Some magnified fluorescent images of F-actin and nuclei of BMSCs and human umbilical vein endothelial cells (HUVECs) on day 30.

As the mechanical properties of 2D, 2.5D, and 3D lattice structures are different, various mechanobiological responses can be expected. In 2D meta-biomaterials, for example, the in-plane properties are more dominant than the out-of-plane properties. These properties can be employed, for instance, to dominate the auxetic behavior of the lattice structure in one direction^57^ or to create hybrid meta-biomaterials^58^ by rationally combining unit-cells with opposite values (i.e., negative and positive values) of the Poisson’s ratio (Figure 2a).

Meta-biomaterials have been assessed for their biocompatibility. For example, Figure 2b^57^ and c^58^ show the adhesion and viability of fibroblast cells and the viability of human mesenchymal stem cells (hMSCs) in contact with meta-biomaterials. Other interactions with cells, such as gene expression, cell morphology, or cell migration, are not extensively studied as of yet.

Our knowledge of the role of the Poisson’s ratio in guiding cell mechanobiology and cell responses when interacting with meta-biomaterials is limited to a few studies. One example is a study in which three different 2D meta-biomaterials with negative, zero, and positive values of the Poisson’s ratio were designed and tested in the presence of mouse bone marrow MSCs (mBMMSCs)^19^ (Figure 2d, top images). However, other geometrical and mechanical properties at the macro-scale (≥cm) were not constant in that study. For example, there was a difference of 310 kPa in the compressive elastic modulus of the PPR (2.63 MPa) and NPR (2.94 MPa) meta-biomaterials. Nevertheless, it was argued that the Poisson’s ratio influences the proliferation and differentiation of mBMMSCs^19^ (Figure 2d, bottom images). Moreover, it was reported that NPR meta-biomaterials exhibit a superior performance as compared to their PPR and ZPR counterparts in terms of cell proliferation and cell differentiation.^19^ More specifically, the most viable stem cells were observed in the NPR scaffolds, followed by those residing in ZPR structures, while the smallest number of cells were found in the PPR specimens. The proliferation assay 3-[4,5-dimethylthiazol-2-yl]-2,5-diphenyltetrazolium bromide (MTT) showed that the proliferation was higher in the NPR meta-biomaterials on days 1, 3, and 5. It was observed that NPR meta-biomaterials promote the differentiation of mBMMSCs into chondrocytes, as evidenced by the expressions of proteoglycans and chondrocyte stromal glycosaminoglycan markers.^19^ Moreover, stem cells could penetrate through the structures, as shown by a cell viability assay imaged by confocal laser scanning microscopy (Figure 2d, bottom images). It is, however, unclear whether the differences between NPR meta-biomaterials and other experimental groups were due to the difference between their Poisson’s ratios or are caused by dissimilarities in the stiffnesses and/or porosity of the meta-biomaterials with different Poisson’s ratios or by the fact that NPR scaffold may better mimic the native soft tissue (i.e., cartilage).

Tuning the local values of the Poisson’s ratio within a 2D meta-biomaterial has been used to control the cellular forces transmitted in regions with different Poisson’s ratios when interacting with embryonic fibroblasts (10T1/2).^45^ The focal adhesion measurements of those cells have shown that the deformations applied by the cells to those meta-biomaterials were larger in the NPR regions as compared to the regions with PPR.^45^ Both regions in the scaffolds showed high cell proliferation. Different cell division patterns were, however, observed in those two regions with unusual cell division occurring for the cells interacting with NPR zones, which may cause genetic instability and potentially lead to cancer.^45^

In addition to 2D (or 2.5D) planar meta-biomaterials, 2.5D cylindrical meta-biomaterials (with an in-plane microarchitecture and an out-of-plane thickness) have shown controllable Poisson’s ratios (Figure 2e).^30^ It has been observed that these structures can also tune the mechanobiological response of the human turbinate MSC (hTMSC).^30^ On day 1, no significant differences were observed in cell densities (i.e., proliferation) between the non-auxetic and auxetic scaffolds (Figure 2f).^30^ On days 4, 7, and 11, however, significantly higher cell proliferation was observed in the auxetic scaffolds. From such microscopical observations, it was concluded that, in the non-auxetic grids, the cells only covered a part of the available surface area, whereas, in the auxetic structure, the cells fully covered the entire area of the scaffold and were strongly interconnected (Figure 2f).^30^ This may be attributed to the geometrical design of the scaffolds given that the interspacing between the struts was smaller in the NPR scaffolds as compared to the PPR ones. Under such conditions, cells may grow and proliferate more easily in the NPR scaffolds.

Although 3D meta-biomaterials can provide a more realistic environment for cells and tissues^59^ to grow, only a limited number of studies have assessed their mechanobiological responses.^22,56^ These studies have analyzed the differentiation of pluripotent stem cells (mouse embryonic stem cells (ESCs) and human induced pluripotent stem cells (hiPSCs)) under interactions with 3D meta-biomaterials^22,56^ (Figure 2g). The first example included two types of auxetic and non-auxetic meta-biomaterials with different Poisson’s ratios as well as different stiffnesses, porosities, and pore sizes. The values were respectively −0.45, 44 kPa, 90.65%, and 250–300 μm for the auxetic meta-biomaterial and 0.3, 100 kPa, 96.31%, and 300–400 μm for the non-auxetic one^22,56^ (Figure 2g). In another example, however, Poisson’s ratios were decoupled from other mechanical properties, including stiffness, resulting in two different auxetic meta-biomaterial designs. The first group had the same Poisson’s ratio (−0.45) but different stiffnesses (i.e., 10 and 94 kPa), while the second group had the same stiffness (almost 100 kPa) but different Poisson’s ratios (0.3 and −0.45). Various differentiation markers, such as β-tubulin III, alkaline phosphatase (ALP), Oct-4, Nanog, CD31, and VE-cadherin, were assayed for neural^22^ and vascular differentiation.^56^ From the biological results of the first category (day 16), the vascular markers CD31 and VE-cadherin were assessed by immunohistochemistry and flow cytometry, and respectively showed 56% and 49% for the auxetic scaffolds. For the non-auxetic scaffolds (in the first category), the vascular markers CD31 and VE-cadherin were 16% and 28%, respectively. It can be concluded that there was more vascular differentiation associated with the cells cultured on the auxetic scaffolds. Similarly, the ALP expression, as an indicator of undifferentiated cells, showed that the non-auxetic scaffolds had higher ALP activity than the auxetic ones. Similarly, the expression of Oct-4 and Nanog was higher for the non-auxetic scaffolds.^56^ As for neural differentiation, it was observed that the auxetic meta-biomaterials upregulated the expression of β-tubulin III marker as compared to the non-auxetic specimens (Figure 2h).^22^ The neural differentiation of mouse ESCs of the second category (i.e., decoupling of the Poisson’s ratio and stiffness) on day 6 showed a similar trend, confirming the role of auxeticity and stiffness in improving neural differentiation (according to the expressions of Nestin, PAX6, and β-tubulin III). This suggests that both NPR scaffolds with higher Poisson’s ratio but similar stiffnesses and NPR scaffolds with higher stiffness but similar Poisson’s ratios promoted neural differentiation.^22^ It is, however, important to note that the increased vascular differentiation and neural expression associated with the NPR scaffolds as compared to the PPR meta-biomaterials may be due to the differences in the pore sizes (i.e., 200–250 μm vs 300–400 μm), pore shapes, porosities, or stiffnesses of both groups.

**Figure 3:**
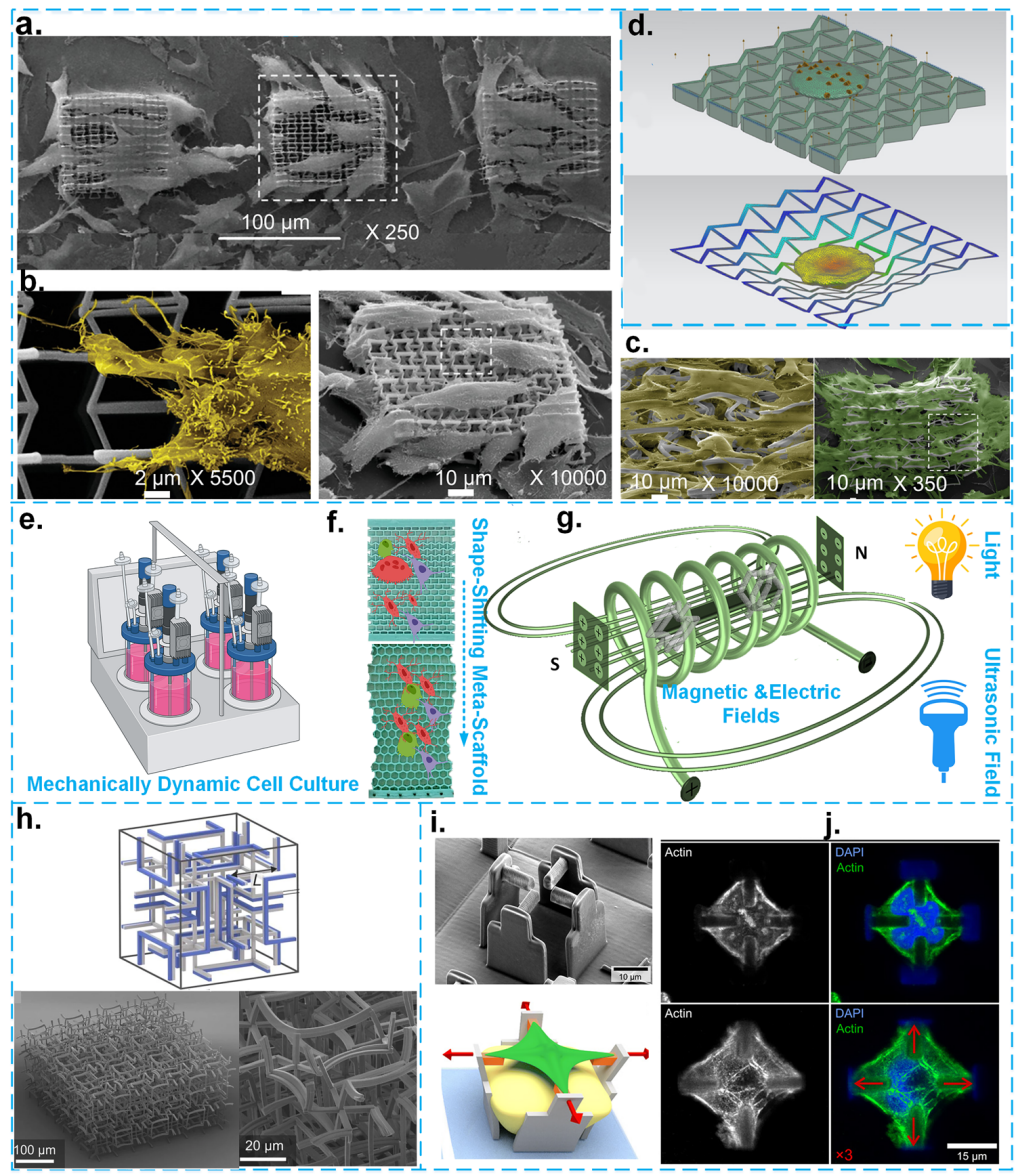
a–c. SEM images of 3D strut-based meta-biomaterials 3D printed by 2PP technique and cultured with fibroblast cell lines at different magnifications. Adapted with permission from ref (20). Copyright 2020 John Wiley and Sons. d. The mechanobiological modeling of the interactions of a single eukaryotic cell with an NPR meta-biomaterial in both deformed and undeformed configurations. Adapted with permission from ref (65). Copyright 2015 IOP Publishing. e–g. A schematic illustration of mechanically (e, adapted with permission from BioRender.com) and remotely (g) dynamic cell culture in meta-biomaterials (f^90^). h. A 4D printed meta-biomaterial at the microscale:^79^ (top) the initial state of the meta-biomaterial (i.e., initial shape); (bottom) the deformed shape of the meta-biomaterial (i.e., temporary shape) under temperature stimulation with two different magnifications. i and j. A 2PP 4D-printed platform with a reversible actuation capability to mechanically stimulate a single cell.^73^ i. A SEM image and a schematic drawing of the platform in the presence of a single cell. j. Some fluorescent images of the F-actin and nuclei of the stretched and unstretched single cells.

The relationship between the pore size and the unit-cell size is of great importance in the design of meta-biomaterials. AM is a promising tool for creating meta-biomaterials at different length scales, from micro- to mesoscales, and with different pore and unit-cell sizes. AM enables the incorporation of more complex features in the design of meta-biomaterials (e.g., Figure 2i and j^60^ and Figure 3a–c^20^). This approach helps in better mimicking the microarchitectural complexity of native (bony) tissue and regulating cell responses at multiple length scales.^60,61^

Porous structures with random microarchitectures can also exhibit an auxetic behavior.^34,62,63^ These structures can be fabricated using either AM or conventional techniques (e.g., foaming).^34,62,63^ The data regarding the biological assessment of meta-biomaterials with random microarchitectures is limited. A rare example is the investigation of the proliferation of an osteoblast-like cell line (MG-63) under static and dynamic loading conditions in the presence of foam-based auxetic scaffolds where the stiffness (via the base material) and degrees of hydrophilicity of the specimens were varied.^34^ The auxetic scaffolds made from polyurethane (PU) promoted the proliferation of chondrocytes between days 3 and 5, which was 1.3 times higher than the non-auxetic specimens.^63^ After day 5, however, there was no significant difference in the proliferation of the cells interacting with the auxetic and non-auxetic scaffolds, likely because 100% confluence was already reached.^63^Table 1 summarizes the reported biological performance and fabrication techniques of strut-based meta-biomaterials, with 2D meta-biomaterials being the most studied structures for such biological analyses.

**Table 1:** Overview of the Current Literature Investigating the Biological Responses of Meta-Biomaterials with Different Values of the Poisson’S Ratio, Scales, Material Properties, Fabrication Techniques, and Cell Types

Scaffold shape	Unit-cell type	Scale	Material	Manufacturing technique	Cell types	ref
2D rectangular	ZPR	meso	polyethylene glycol (PEG)	AM-pSLA	hMSCs	(91)
	PPR/NPR					(58)
3D rectangular	NPR	micro/meso	organic–inorganic hybrid SZ2080	AM-2PP	mouse fibroblast cell	(20)
2D rectangular	NPR	micro/meso	Polycaprolactone (PCL)	MEW	HUVECs and BMSCs	(60)
2D rectangular	PPR/NPR	meso	BR-7432IG30 polyaliphatic urethane acrylate blend	AM-pSLA	fibroblast	(57)
2D rectangular	PPR/NPR	micro	-	DLP	-	(45)
3D rectangular	PPR/NPR	-	Polyurethane	A compressed carbon dioxide assisted technique	ESCs) and hiPSCs.	(22,56)
2.5D tubular	NPR	meso	PCL	MEW	-	(92)
2D circular	PPR/ZPR/NPR	meso	CNF/PEGDA aerogel	SLA and freeze-drying	mBMSC	(19)
2D rectangular	NPR	micro	silicon	DRIE	hMSCs	(65)
3D rectangular	NPR	macro	HA/PGLA and PU	solvent casting/salt leaching	MG-63	(34,62,63)
2.5D tubular	PPR/NPR	micro	PEGDA	AM-pSLA	hTMSCs	(30)

In addition to in vitro studies on meta-biomaterials, several works have focused on computational modeling and optimization of bone scaffolds with respect to their mechanobiological responses.^64^ More specifically, in auxetic meta-biomaterials, the interaction between a single eukaryotic cell and a 2D auxetic meta-biomaterial has been modeled.^65^ This model has been employed to design a cell-growth sensor to measure the forces applied by cells to the auxetic scaffold (Figure 3d). More interestingly, the presence of the cells can also change the mode shapes of the scaffold and even their orders of appearance.^65^

#### Meta-Biomaterials under Dynamic Conditions

To effectively mimic the microenvironments of tissues and the homogeneous distribution of cells within scaffolds, it is important to consider the impact of the dynamic behavior of either meta-biomaterials (i.e., dynamic loading condition) or the environment (i.e., dynamic environments). Indeed, in the body, the dynamic microenvironment surrounding cells continually regulates different cell functions, such as differentiation and proliferation. To better mimic these conditions, dynamic cell cultures need to be employed.^66^ There are several factors that can improve cell proliferation and differentiation under dynamic cell culture conditions. Dynamic cell cultures provide mechanical forces that resemble those found in native tissues, thereby enabling a transition between biochemical and biomechanical cues. They also create a uniform cell distribution and establish a dynamic supply of nutrient to cells.^67−900^ Another benefit of using dynamic cell cultures is that they allow for guiding cell growth in the scaffolds in a specific (confined) environment. To clearly elucidate the effects of the Poisson’s ratio of meta-biomaterials on cell response, dynamic loading conditions must be applied. It is, therefore, important to know how dynamic cell cultures work and to implement this approach in future research to better understand the living cell–meta-biomaterial interactions. Moreover, the biodegradation rate of scaffolds depends on the type of loading and may be different under dynamic loading conditions as compared to static conditions.^21^ The biodegradation rate of scaffolds should match the deposition rate of the newly formed ECM to maintain a balance between the degradation and formation of new tissue.^21^

There are generally two methods to operate a dynamic cell culture: mechanically induced loading (e.g., mechanical bioreactors) and remotely induced (e.g., magnetic/electric field or ultrasonic field) actuation^68^ (Figure 3e–g). Although auxetic behavior is more dominant under dynamic loading, only a few studies have investigated simultaneous mechanical loading and cell culturing of meta-biomaterials.^34,62^ A foam-based auxetic scaffold is the only example that was tested under dynamic cell culture conditions. The results of that study showed a higher proliferation of MG-63 osteoblast-like cells (i.e., 200% for the stiffer scaffold and 20% for the softer one) under dynamic cell culture conditions.^34^ There is, however, no example of a remotely induced dynamic cell culture platform testing the mechanobiological response of meta-biomaterials.

## Micro-AM Technology to Fabricate Meta-Biomaterials

Over the past years, AM technology has matured enough to create meta-biomaterials with reliable and reproducible properties that can mimic some of the biological and mechanical characteristics of the native bony tissue. The progress of AM techniques has paved the way for creating meta-biomaterials with complex microarchitectures, thereby enabling the creation of a platform to effectively assess the role of microarchitectural features, such as auxeticity and local curvatures, in (bone) tissue engineering processes.

Light-assisted AM techniques have, so far, been the most widely used 3D printing methods to create meta-biomaterials at the microscale. This is due to the availability of a wide range of materials (i.e., biocompatible polymers) and the ability of these techniques to print at very high resolutions with minimum feature sizes in the micron range.^59,70^ Examples of these techniques are stereolithography (SLA)^30,57^ and two-photon polymerization (2PP).^20,71,1000^ Different meta-biomaterials with 3D multiscale features and sizes down to submicron ranges have been 3D printed using 2PP.^72−73^ The 2PP AM technique, like other similar light-assisted techniques, can be combined with conventional manufacturing techniques (e.g., molding) in a hybrid fashion to push the boundaries of the existing 3D printing techniques. This approach has been used, for example, to study the curvature-dependent mechanobiology of bone cells at the microscale, by integrating molded 2PP 3D printed structures and creating soft elastomeric microsurfaces.^74^ This approach can be further extended to develop meta-biomaterials with tunable morphological and material properties in the future.

One challenge in creating microscale meta-biomaterials is the trade-off between the printing time and print quality, particularly when covering multiple length scales. A higher degree of geometrical complexity often translates to a longer fabrication time and more complex postprocessing steps. In addition, biocompatibility, biodegradability, and printing throughput are the most challenging aspects of microfabrication, particularly for 2PP.^75^ In future studies, stimuli-responsive materials, such as magneto-responsive materials, can be used to create meta-biomaterials with dynamic and tunable properties.^76^

## Future Research

Here, we have reviewed the current progress of meta-biomaterials, their corresponding biological assessments, and the relevant mechanobiological pathways. We specifically focused on how the different values of the Poisson’s ratio (i.e., the degree of auxeticity), which is an indication of the geometrical properties of lattice structures, can influence the biological responses of meta-biomaterials. In addition, we highlighted the importance of dynamic cell culturing and its effects on (bone) tissue engineering using meta-biomaterials. In this section, we discuss the outlook and future directions of this research line and provide several suggestions for follow-up studies.

### Outlook and Future Work

Auxeticity, as a “mechanobiological tool” for the development of the next generation of meta-biomaterials, can fine-tune the bone regeneration process. Several studies dealing with the effects of the Poisson’s ratio on the mechanobiological response of meta-biomaterials have already appeared in the literature. However, more studies are needed to elucidate the isolated effects of the Poisson’s ratio on the cell response. That is because the Poisson’s ratio and other geometrical and mechanical properties of meta-biomaterials are highly inter-related. Extreme care, therefore, needs to be taken to ensure these factors are separated from each other to the maximum possible extent.

Another missing aspect in the current body of literature is the effects of the Poisson’s ratio on the responses of cells in 3D meta-biomaterials. The variations in the configuration of struts in 2D and 3D structures may cause notable differences in the response of cells interacting with such meta-biomaterials. Therefore, the mechanobiological results of 2D and 2.5D meta-biomaterials cannot necessarily be extrapolated to the 3D ones. To date, only a limited number of studies have addressed the role of the Poisson’s ratio in 3D meta-biomaterials.^22,56^ Further investigations are, therefore, required to understand any such differences between 2D and 2.5D structures on the one hand and 3D structures on the other.

From a biological viewpoint, only a few cell types have been so far used to assess the potential of meta-biomaterials in (bone) tissue engineering. Further research with different cell types (i.e., either cell lines or primary cells) is, therefore, required under both monoculture and coculture conditions. Moreover, most of the biological assessments performed on meta-biomaterials are limited to the assessment of their cytocompatibility and cell proliferation. Other biological assays are, thus, required to investigate the effects of meta-biomaterial properties on the differentiation of cells. In addition, in vivo experiments are needed to allow for the implementation of meta-biomaterials in clinical settings.

From a manufacturing viewpoint, it remains challenging to create multiscale meta-biomaterials at different length scales with high throughput. Recent developments in multimaterial AM have provided new opportunities for incorporating more complexity in the design of meta-biomaterials through the deposition of soft and hard materials.^15^ This may help in decoupling the Poisson’s ratio from other mechanical properties, thus providing additional flexibility in the design of meta-biomaterials. Moreover, organic–inorganic hybrid materials^77^ can be used to independently tune the elastic modulus and mechanical performance of meta-biomaterials along with their Poisson’s ratio. These materials can be 3D printed at the microscale, providing precise control over their microarchitectural features and offering a promising avenue for the development of advanced engineered microenvironments with multifaceted functionalities for various biomedical applications.

In addition, it is still unclear how meta-biomaterials can stimulate cell response under dynamic loading conditions. Meta-biomaterials can show rare properties under external loading, such as local shape-morphing, which can be tuned by varying the Poisson’s ratio of individual unit-cells (Figure 3h).^78−80^ However, not much is known about how these unique features can influence the cell response. Although it has been shown that external stimuli, such as magnetic or electric fields, light, and ultrasound, may improve new tissue formation^81,1900^ or facilitate in vitro studies,^82^ their effects in connection with meta-biomaterials remain elusive. It is also not quite clear how these external stimuli can trigger other biochemical/biological activities in cells and alter their gene expression.^68,73^ One example of such systems is a 4D printed reversible scaffold designed to mechanically stimulate single cells with the aim of altering their gene expressions (e.g., Figures 3i and j^73^). More studies are needed to explore the response of cells to the meta-biomaterials stimulated by either mechanical loading or by other types of external stimulus.

4D (bio)printing is a promising AM technology to study the dynamic properties of meta-biomaterials and their cell responses. Creating 4D-printed meta-biomaterials (i.e., structures changing their shape with time^83^) with auxetic properties may be a new research direction to promote tissue formation and influence the response of cells to such types of biomaterials. This approach can provide additional functionality for the design of meta-biomaterials, for example, to create medical devices with integrated drug delivery systems providing certain antimicrobial activities.^84^ 4D-printed medical devices have many applications ranging between cardiovascular engineering^67^ to orthopedic implants^85^ and beyond to create specific biological responses.^73^ One recent example of such applications involves the development of stimuli-responsive deployable metamaterials with dynamic Poisson’s ratios (Figure 3h).^79,86,2100^ Moreover, 4D-printed deployable implants can be implanted using minimally invasive surgical techniques. Upon external actuation or stimulation, such deployable meta-implants expand and fit into a cavity or defect zone.^87,2200^ It is, however, important to gain a better understanding of the interaction between 4D-(bio)printed structures and living tissues. For example, 4D (bio)printing technology can be used to control the orientation of hMSCs, human embryonic stem cell-derived cardiomyocytes, and endothelial cells in a light-responsive cardiac construct.^78^ Finally, to comprehensively understand the dynamic mechanobiology of meta-biomaterials, follow-up studies on implementing 4D-printed meta-biomaterials as microrobots^79,80,88,2300^ can be conducted in the future.

The lack of multiphysics computational models for simulating the mechanobiological response of meta-biomaterials and their interactions with living cells is another challenge in this field. Such in silico models represent powerful tools for designing optimal meta-biomaterials with the aim of reducing the cost and time associated with such studies. These models, when coupled with bone modeling approaches,^89,2400^ can serve as an effective tool to predict the mechanical behavior resulting from various microarchitectural designs of meta-biomaterials. They can be also used to better understand the mechanobiological events (e.g., force transmission) occurring within different cell compartments (e.g., nuclei and cytoplasm).

## Conclusions

Although it has been shown that biophysical cues, such as mechanical properties (e.g., stiffness) and geometrical properties (e.g., pore size and porosity), are among the most important parameters to successfully design orthopedic implants, there is a lack of understanding as to how microarchitectures influence the bone tissue regeneration process. One parameter than can widely vary depending on the microarchitectural design is the Poisson’s ratio. In particular, it has been shown that the sign of the Poisson’s ratio (i.e., negative or positive values) may play a notable role in guiding force transmission across cells, while also affecting the cell response in terms of cell proliferation, adhesion, differentiation, and directionality. Here, we have discussed the current state of the art regarding the Poisson’s ratio-driven meta-biomaterials and their effects on cell-biomaterial interactions.

Auxetic behavior has been observed in native (soft and hard) tissues and cells, highlighting its importance in designing the next generation of scaffolds and implants. In order to effectively design architected biomaterials inspired by native tissues, it is essential to consider not only the stiffness and microarchitectural parameters of such biomaterials (e.g., local curvature, porosity, and pore size) but also their Poisson’s ratio. There is some evidence in the literature suggesting that NPR meta-biomaterials promote proliferation and differentiation of cells in vitro. It is, however, necessary to decouple the effects of the Poisson’s ratio from other geometrical and mechanical properties. Moreover, most current studies are limited to 2D meta-biomaterials, and needs to be extended to 3D variants.

The concept of auxeticity assumes an even more interesting role within dynamic loading conditions. Advanced technologies, such as 4D (bio)printing technologies, have shown great promise in creating such meta-biomaterials with dynamic properties. This requires the use of stimuli-responsive biomaterials and a further analysis of the response of living cells to 4D-printed meta-biomaterials. Future studies of such novel effects call for interdisciplinary approaches in which engineers and scientists from various backgrounds, such as mechanical engineering, biology, physics, and materials science, work together to achieve a better understanding of the mechanobiological pathways driving the response of cells to auxetic and non-auxetic meta-biomaterials.
